# Nasal mucosal reactivity assessment via a double-blind placebo-controlled food challenge with cow’s milk allergens

**DOI:** 10.1186/s13223-022-00700-3

**Published:** 2022-07-01

**Authors:** Edyta Krzych-Fałta, Oksana Wojas, Piotr Samel-Kowalik, Adam J. Sybilski, Bolesław Samoliński

**Affiliations:** 1 Department Basic of Nursing Faculty of Health Sciences, Medical University of Warsaw, Warsaw, Poland; 2 Department of Prevention of Environmental Hazards, Allergology and Immunology Faculty of Health Sciences, Medical University of Warsaw, Warsaw, Poland; 3 2nd Department of Paediatrics, Centre of Postgraduate Medical Education, Warsaw, Poland; 4 Department of Paediatrics and Neonatology with Allergology Center, Central Clinical Hospital of the Ministry of the Interior, Warsaw, Poland

**Keywords:** Food allergy, Cow’s milk allergy, Placebo-controlled food challenge, Nasal mucosal reactivity

## Abstract

**Background:**

Allergies, including food allergies, are a considerable clinical and public-health problem. The introduced preventive measures and differential diagnostics, including oral food challenges, are the gold standard for determining further treatment planning.

**Case presentation:**

We present a case of an 18-year-old girl with a cow’s milk allergy who underwent an oral food challenge (double blind oral food challenge). Such a challenge may be confounded by inducing a response from other systems and organs, which provides theoretical grounds for the use of other methods of assessing the body’s response to food allergens (response demonstrated by the upper respiratory tract). Based on this idea, in order to assess the degree of mucosal response, we used optical rhinometry as an objective method for nasal patency evaluation, as well as identification of tryptase level in nasal lavage fluid and exfoliative cytology of nasal mucosa. The results of these tests confirmed positive reaction of the nasal mucosa in the course of the oral allergen challenge.

**Conclusions:**

The observed increase in the nasal mucosal reactivity that accompanies oral food challenges may suggest a potential for using food allergens in nasal allergen provocation testing in order to diagnose food allergies.

## Background

Diagnosing food allergies is a difficult and tedious process due to the great variety of symptom-triggering factors and a variety of the symptoms themselves. The standard management in patients with a suspected food allergy is based on a detailed history, physical examination, skin tests (skin prick tests, prick by prick tests, and atopy patch tests), laboratory tests [sIgE, component-resolved diagnostics (CRD), basophil activation test (BAT)], and elimination diets. However, a double-blind placebo-controlled food challenge (DBPCFC) still remains the most important investigation and the gold standard in food allergy diagnostics [1, 2]. The main purpose behind DBPCFC is the need to confirm the causal relationship between the consumption of a certain food and the subsequent hypersensitivity reaction. This test to some extent recreates and mimics the body’s natural response to the given food. A positive result is typically the determining factor for introducing an elimination diet. One important aspect is the fact that every DBPCFC conducted for diagnostic purposes carries the risk of inducing bothersome or dangerous symptoms. Considering the risk of anaphylaxis, any DBPCFC testing should be conducted in a hospital setting [3, 4]. The clinical presentation of food allergies is exceptionally diverse and depends on the type of food, patient age, and individual predisposition. Undoubtedly the most common manifestations are gastrointestinal symptoms, which may come from any segment of the gastrointestinal tract—spanning from the oral cavity (aphthous stomatitis) through the esophagus (eosinophilic esophagitis) and stomach (epigastric pain, nausea, vomiting) to the small and large intestines (enteropathies, eosinophilic enterocolitis) [1, 4]. Other food allergy manifestations include oral allergy syndrome, anaphylactic shock, urticaria, atopic dermatitis, and contact dermatitis. Interestingly, the symptoms of allergic rhinitis and asthma may be also caused by a food allergy [3, 4]. Moreover, oral food challenges have been associated with nasal symptoms, such as itching, sneezing, watery nasal discharge, and nasal congestion [1–3]. These observations have been the cornerstone for studies on the use of nasal allergen provocation testing in food allergy diagnostics. Nasal allergen provocation tests are widely used in diagnosing rhinitis, since they show causality, help identify the triggering factors of IgE-mediated nasal hypersensitivity reactions, and confirm the efficacy of medication and allergen-specific immunotherapy in the treatment of allergic rhinitis. Moreover, nasal allergen provocation testing plays an important role in diagnosing local allergic rhinitis and in the differential diagnosis of various types of rhinitis. Nasal allergen provocation testing is a relatively safe procedure, so it may be conducted in an outpatient setting. Any immediate hypersensitivity reactions usually resolve spontaneously within twenty minutes. The results of a nasal allergy provocation test are interpreted in conjunction with the reported symptoms and objective assessments, such as rhinomanometry, acoustic rhinometry, peak nasal inspiratory flow (PNIF), and optical rhinometry [5, 6].

We present the case report of a female patient who underwent a placebo-controlled food challenge and whose local nasal mucosa response was measured with subjective (Total Nasal Score) and objective techniques for assessing nasal obstruction (optical rhinometry and immunoenzymatic assays for measuring tryptase levels in nasal lavage fluid). A clear positive reaction of our patient’s nasal mucosa observed in the course of oral milk allergen challenge demonstrates the potential of intranasal allergen challenges to be used as valuable markers in the process of diagnosing food allergies.

### Case presentation

In the year 2021, an 18-year-old female presented at the Allergy Consultation Clinic due to an approximately 10-year history of episodes of abdominal pain and nausea following the consumption of milk-containing products. The patient was a high school student, who was born and lives in a large city. She denied any allergic symptoms in her infancy and early childhood. She was breastfed until the age of 12 months, and reported that neither cow’s milk nor dairy products produced any abdominal discomfort until the age of 8 years. The patient’s mother was diagnosed with cow’s milk allergy a number of years previously and has been on a milk-free diet ever since. No other members of the patient’s family have allergies. Three years earlier, the patient developed abdominal pain, nausea, vomiting, vertigo, weakness, and generalized itchiness approximately 15 min after drinking a glass of warm cow’s milk. At that time, the patient took an antihistamine agent, whose name she cannot remember, and her symptoms resolved 40 min later. The patient did not seek medical attention or visited the Allergy Consultation Clinic. After that episode, the patient limited only the consumption of milk in her diet, but experienced abdominal discomfort following the consumption of cheese, cottage cheese, and yoghurts. Moreover, for the last three years at the end of March and in April, the patient had been experiencing nasal congestion, itching, and watery discharge. She denied cough and wheezing. The patient reported being generally healthy and denied any chronic diseases and long-term medication.

### Physical examination findings and differential diagnostics

The patient underwent a complete physical examination at the Clinic, including a thorough otorhinolaryngological examination. The findings included no relevant abnormalities apart from dry skin and mild inferior turbinate hypertrophy. She also underwent skin prick tests (*Alleropharma*) with inhaled and food allergens. Positive results were observed for the allergens of cow’s milk 8/16, goat’s milk 4/7, cod 10/20, and birch 10/20, with positive control (histamine) 5/10 and negative control 0/0. Serum allergen-specific IgE levels were also measured (food and inhaled allergen panel) and yielded grade 4 levels of cow’s milk-specific IgE and grade 3 levels of goat’s milk-specific IgE, with negative results for other allergens. CRD revealed alpha-lactalbumin (Bos d 4) levels of 0.63 FIU/mL, beta-lactoglobulin (Bos d 5) levels of 6.12 FIU/mL, and casein (Bos d 8) levels of 22.23 FIU/mL. The material for exfoliative cytology examination of nasal mucosa was collected from the right inferior nasal concha, 1 cm from its anterior edge, using a disposable inoculation loop (nasal curette). The collection technique involved repeated rubbing of the nasal mucosa from its posterior segment towards the anterior segment (scraping method), followed by quick spreading of the material on a microscope slide. After that, the material was fixed with Cytofix aerosol (manufactured by Sanko). Hematoxylin and eosin staining was conducted. The specimen was then assessed using a Delta Microscope Optical Evolution 300 microscope at 400 × magnification. Exfoliative cytology of the nasal mucosa revealed columnar epithelial (61.0%), goblet (5.0%), basal (5.0%), and squamous (62.1%) cells and neutrophils (16.9%). No eosinophils were detected. Additionally, a hydrogen breath test was conducted to exclude lactose absorption problems; the test was negative.

### The course of the oral food challenge with cow’s milk allergens accompanied by nasal mucosal response monitoring

The patient, who was in good general condition, underwent the test after the recommended fasting period and having provided her written informed consent (KB 65/2021). This work has been financed by the Medical University of Warsaw grant no. PW/Z/2/2/20(1). The assessment was planned in accordance with the Polish Society for Pediatric Gastroenterology, Hepatology, and Nutrition food allergy division recommendations [7] as well as the *European Standard and PRACTALL guidelines* [8, 9]. The double-blind food challenge was conducted as follows: ¼ of a muffin, ¼ of a muffin, ¼ of a muffin, ¼ of a muffin, and a whole muffin in 15-min intervals. The active allergen and placebo, which were identical in appearance and taste, had been prepared by a dietician. The muffins had been prepared according the following recipe: (Active product) 250 g of wheat flour, 10 g of baking powder, 25 g of sugar, 50 mL of rape oil, 250 mL of cow’s milk, 1 teaspoonful of vanilla extract, and a pinch of salt. The ingredients were mixed and the mixture was transferred into a baking form and baked for 15 min at 180 degrees Celsius. The quantity of milk per portion was 12.5 mL in a ½ of a muffin and 25 mL in a whole muffin. (Placebo product): 250 g of wheat flour, 10 g of baking powder, 25 g of sugar, 50 mL of rape oil, 250 mL of soy milk, 1 teaspoonful of vanilla extract, and a pinch of salt. The ingredients were mixed and the mixture was transferred into a baking form and baked for 15 min at 180 degrees Celsius. The ingredients in these recipes yielded 10 muffins each.

Four weeks prior to the challenge, the patient was put on an elimination diet, with no cow’s milk products. During that period, the patient used no antihistamines or corticosteroids in any form. The patient’s body weight was 61 kg, height 164 cm. The challenge was conducted by qualified personnel with access to an anaphylaxis kit, in a hospital setting, and outside the birch pollen season. On the day of the challenge, the patient was healthy with no evidence of infection. The physical examination revealed no relevant abnormalities. After each muffin portion was consumed, the patient’s general condition, pulse, blood pressure, and skin were assessed and her chest was auscultated. Blood pressure was 120/70, pulse 72/min, oxygen saturation 99%, the skin was clear, and auscultation revealed normal breath sounds. For organizational reasons the challenge was conducted in two stages 3 h apart. During the first stage, there were no gastrointestinal, dermatological, or respiratory symptoms or any changes in pulse, blood pressure, or oxygen saturation. The patient’s general condition was excellent. Fluctuations in nasal patency, including the physiological nasal cycle, were present throughout the duration of the challenge, with the characteristic variability in nasal cavity diameters. The second stage of the challenge was conducted three hours after the first one. The measurements were conducted in real time during the oral food challenge, and the results were recorded in the assessment report. The nasal obstruction status was assessed every 15 min, in accordance with both oral food challenge and nasal provocation test protocols. The assessment was resumed according to the adopted protocol: after administering ¼ of a muffin, ¼ of a muffin, and ¼ of a muffin (a total of ¾ of a muffin) there were no clinical manifestations; however, seven minutes after the next portion of ¼ of a muffin (one whole muffin in total), the patient reported nausea and abdominal pain. A slight increase in the pulse rate up to 85/min was noted, blood pressure was 110/75, oxygen saturation 99%. She developed mild erythema on the skin of her cheeks without urticaria, dyspnea, or weakness. The abdomen was soft and nontender on palpation; bowel sounds were hyperactive. After another 10 min, the abdominal pain and nausea became exacerbated. The challenge was discontinued, and the result was considered to be positive. Unblinding revealed that the symptoms developed following the administration of one whole muffin containing 25 mL of cow’s milk (active sample). After another 15 min of follow-up, the symptoms gradually subsided and eventually resolved completely.

**Fig. 1:**
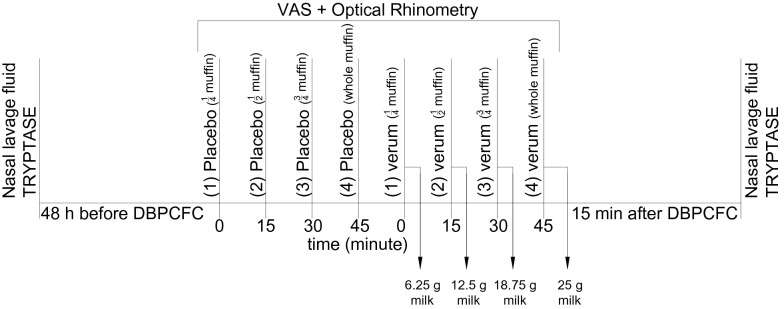
Study design

**Fig. 2:**
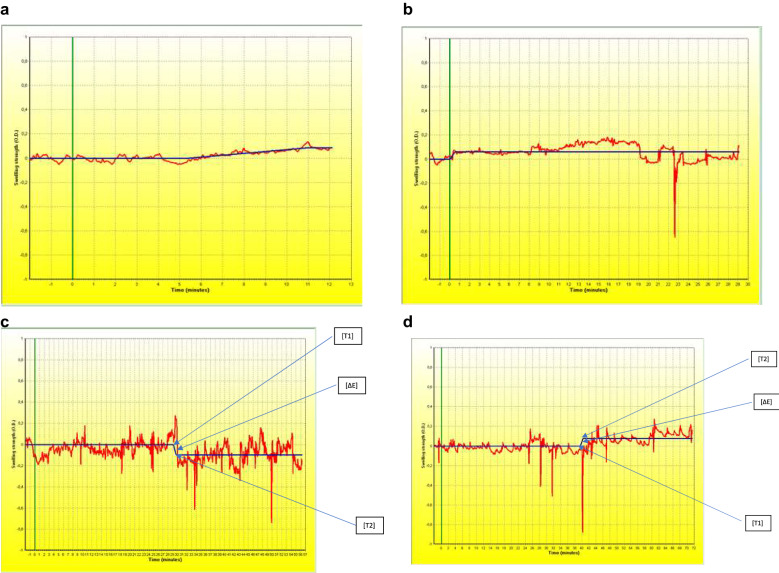
Nasal mucosal response in the oral food challenge (placebo). axis X-length of time in general, axis Y-optical density measured in OD, ΔE-optical density, T1-the beginning of the reaction (time), T2-time to achieve the highest response in the nasal cavity membrane. **a** The first assessment–placebo; ΔE = − 0.08 OD, T1 = 321 s (5:21), T2 = 658 s (10:58). **b** The second assessment–placebo; ΔE = − 0.06 OD, T1 = 3 s (0:03), T2 = 32 s (0:32). **c** The third assessment–placebo; ΔE = − 0.10 OD, T1 = 1752 s (29:12), T2 = 1787 s (29:47). **d** The fourth assessment–placebo; ΔE = − 0.08 OD, T1 = 2377 s (39:37), T2 = 2422 s (40:22).

**Fig. 3:**
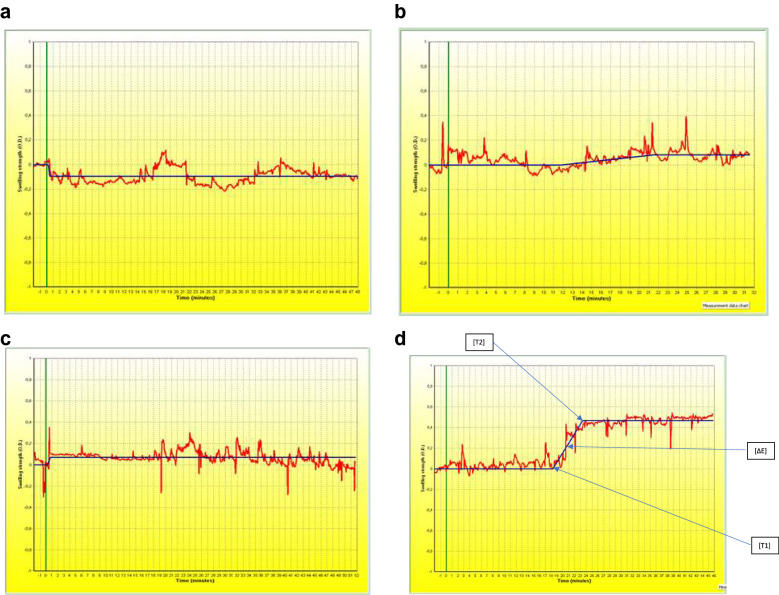
Nasal mucosal response to an oral food challenge with cow’s milk allergens. axis X-length of time in general, axis Y-optical density measured in OD, ΔE-optical density, T1-the beginning of the reaction (time), T2-time to achieve the highest response in the nasal cavity membrane. **a** The first assessment in the oral food challenge with cow’s milk allergens–(¼ of a muffin); ΔE = − 0.10OD, T1 = 15 s (0:15), T2 = 30 s (0:30). **b** The second assessment in the oral food challenge with cow’s milk allergens – (½ of a muffin); ΔE = − 0.08 OD, T1 = 709 s (11:49), T2 = 1287 s (21:27). **c** The third assessment in the oral food challenge with cow’s milk allergens–(¾ of a muffin); ΔE = − 0.07 OD, T1 = 12 s (0:12), T2 = 45 s (0:45). **d** The fourth assessment in the oral food challenge with cow’s milk allergens–(one whole muffin); ΔE = 0.47 OD, T1 = 1098 s (18:18), T2 = 1402 s (23:22)

Simultaneously with the food challenge, nasal obstruction was assessed subjectively, with a Total Nasal Score, and objectively, with optical rhinometry (emission spectroscopy, GmbH Rhios, Groerkmannsdorf, Germany) and nasal lavage fluid tryptase (UniCAP, Sweden), which is considered a specific marker of mast cell activation (Fig. 1 Study design). The optical rhinometer is equipped with an optical sensor and a light emitter placed across the bridge of the nose. The light emitter generates 0.2 s pulses of infrared light with the mean wavelength of 600–800 µm, and the sensor continuously and directly measures changes in nasal airway patency (changes in the extent to which the assessed medium slows down and scatters the light beam; in other words optical density (OD) expressed as ∆E [10, 11]). Nasal lavage fluid, collected with Greiff’s technique [12], was centrifuged at 1,000 rpm for 15 min in preparation for an immunoenzymatic assay to detect tryptase, with a sensitivity threshold of 1.0 µg/L. Nasal fluid was collected twice: eight hours prior to the scheduled challenge, in order to minimize the risk of nasal mucosal over reactivity, and after the local nasal mucosal response in the cow’s milk food challenge, which was at hour 2 of the assessment. Nasal lavage fluid was collected with the use of a specially designed tool equipped with two tubes: one for saline administration (administered at room temperature, 8 mL to each nasal opening) and the other for the draining of nasal lavage fluid. Nasal irrigation was performed twice and the obtained biological material was then subjected to further laboratory tests. The subjective and objective assessments were conducted separately, four times, during the placebo phase and after cow’s milk allergen administration, in accordance with the protocol. Optical rhinometry was used to assess the onset of nasal mucosal response (T1), time of maximum response (T2), and optical density. Apart from these objective assessment techniques, a subjective assessment tool was also used (Total Nasal Score). Our optical rhinometry assessments revealed considerable fluctuations in the recorded blood flow during the oral food challenge. Interestingly, there was a significant increase in optical density (up to 0.47 OD) following a cumulative administration of 25 g of cow’s milk, which corresponds to a positive nasal allergen provocation test result (Fig. 2 Nasal mucosal response in the oral food challenge (placebo), Fig. 3 Nasal mucosa response to an oral food challenge with cow’s milk allergens). Tryptase levels in nasal lavage fluid were 278 µL at baseline and 386 µL after the challenge. Changes in the nasal patency recorded during the oral food challenge with cow’s milk allergens were additionally accompanied by upper respiratory symptoms in the form of nasal itching (2 points in a 0–3 point scale) and sensation of nasal obstruction (2 points in a 0–3 point scale). These symptoms were absent during the placebo stage of the oral food challenge. No other relevant symptoms were noted during the assessment.

The patient was followed up for 2 more hours and discharged home in good general condition. Three days after the assessments, another exfoliative cytology of the nasal mucosa was performed and revealed the presence of eosinophils (53%), columnar epithelial cells (15%), squamous epithelial cells (30%), and neutrophils (12.3%). Due to the positive result of the oral food challenge with cow’s milk allergens, the patient was recommended a milk-free diet and was referred to a dietician, with a view to designing a balanced diet.

## Discussion

This paper is a case report on a patient with cow’s milk allergy, whose diagnostic assessments included measuring nasal mucosa reactivity in addition to performing a standard oral food challenge. Moreover, the objective investigations included optical rhinometry, which is another factor that makes our study unique. Optical rhinometry offers a wide range of options in assessing nasal patency changes; this includes the use of this technique in allergen provocation testing. As demonstrated by literature review, the positive result of the challenge measured via the objective nasal patency assessment technique—optical rhinometry—was 0.2 OD [13]. Therefore, we can conclude that nasal mucosa response to the oral food challenge, which was assessed in our study, yielded a positive result, which undoubtedly supports expanding the current indications for nasal allergen provocation testing [6].

Food allergy is a combination of symptoms developing after each exposure to a given food at a dose that is tolerated by healthy individuals. Unlike food intolerance, food allergy is an adverse, IgE-mediated or non-IgE-mediated, immune reaction to a certain food [2, 11]. The pathophysiology of food allergies involves complex interactions between the gut mucosa, local and systemic immunity, and the microbiome. The prevalence of food allergy has been increasing worldwide, which makes it a serious public health problem. The current estimated prevalence of food allergy at 6–10% in children and 2–5% in adults. An estimated over 220 million people worldwide suffer from a food allergy. Although there are no precise epidemiological studies, the prevalence of food allergies in Western countries seems to have increased considerably over the last two decades and is currently approximately 10% in preschool children [14]. Food allergens are primarily glycoproteins with a molecular weight of 15–50 kD. The allergenicity of molecules depends on the number of epitopes capable of binding specific antibodies, with epitope structure determining the persistence or loss of allergenic properties [14, 15]. The gut mucosa is permeable to protein and carbohydrate molecules found in the gut lumen. Approximately 90% of proteins from the gastrointestinal tract are believed to undergo transcytosis, with 10% of them transferring across gastrointestinal walls unchanged. The immune barrier formed by gut-associated lymphoid tissue (GALT) and non-immune barriers (gastric juice, gastrointestinal enzymes and hormones) jointly prevent allergens from penetrating the gut mucosa and entering the bloodstream. The combined effects of these barrier types lead to immune exclusion, immune elimination, and the development of immune tolerance [15]. A pathological response leads to the production of specific antibodies against food allergens and the formation of immune memory cells. Another, subsequent contact with the allergen activates reactions leading to mast cell and basophil degranulation and the release of mediators of multidirectional biological effects (histamine, tryptase). Undoubtedly, the changes in nasal fluid tryptase levels evaluated in our study are a specific response to immune reaction mediators, including those in the nasal cavity, over the course of the oral food challenge. Exfoliative cytology of the nasal mucosa performed three days after the challenge showed an increased proportion of eosinophils, which were absent at baseline. These findings support the usefulness of assessing nasal mucosa reactivity during an oral food challenge.

Allergy to cow’s milk is defined as a repetitive adverse immune reaction to the consumption of foods containing milk; this reaction can be IgE-mediated, non-IgE-mediated, or mixed [16, 17]. The clinical presentation varies widely and involves the gastrointestinal, respiratory, integumentary, and other systems. The most common of the sensitizing cow’s milk proteins are casein, beta-lactoglobulin and alpha-lactalbumin. The prevalence of allergies to milk ranges from 2 to 5% depending on the population. Most patients develop food tolerance by the age of 3 years. However, persistent allergy affects 20% of patients after 16 years of age [16, 17]. The most reliable assessment method in the diagnostics of cow’s milk allergy is an oral food challenge, which plays a particularly important role in confirming a milk allergy and establishing an elimination diet, as well as excluding other conditions, which require a different management. However, decisions to perform a challenge should take into account the limitations of this method, which include the risk of anaphylaxis or another severe reaction, the possibility of obtaining false negative or false positive results, and the cost associated with conducting oral food challenges at a hospital setting [16, 17]. Because of these considerations, there is a need to search for safer, more accessible diagnostic techniques that can be performed in outpatient settings. In 2013, Kvenshagen and Jacobsen emphasized the necessity of novel diagnostic methods for food allergies. This was a result of the increased incidence and the risks, the high costs, and the time-consuming nature of oral food challenges [18]. Their review of medical literature yielded the possibility of using the mucosal allergen challenge (i.e., endoscopically guided nasal, conjunctival, and labial challenges) in food allergy diagnostics. Consequently, they considered mucosal allergen challenge techniques promising, due to an easy access to mucous membranes and the possibility of using low allergen doses. In 1985, Amlot et al. conducted a study with the use of nasal, labial, and gastric challenge in 39 patients with milk and egg allergy diagnosed based on the history and positive skin prick test results. The result of nasal allergen provocation tests were interpreted based on PNIF measurements and the number of sneezes. No oral food challenge was conducted. Based on the results, nasal allergen provocation testing was considered the most sensitive method. Nonetheless, there have been few studies demonstrating the use of nasal allergen provocation testing in the diagnosis of food allergies [19]. Seppey et al. [20] and Clark et al. [21, 22] published their studies showing the use of nasal allergen provocation testing with egg and peanut allergens. Those authors used facial thermography to assess the results and concluded that such provocation testing was rapid, safe, and objective. Gelis et al. conducted an interesting study in which they assessed the usefulness of nasal allergen provocation testing as an alternative to an oral food challenge in diagnosing allergy to shellfish and in differentiating an allergy from non-allergic hypersensitivity. The study was conducted in 45 patients with a shrimp allergy confirmed via a skin test, oral food challenge results, a past anaphylactic episode, or a history of shrimp intolerance. The control group consisted of 10 healthy individuals. The allergen used in the nasal provocation test was a lyophilisate of boiled shrimp, and the results were assessed with the help of acoustic rhynometry and a visual analog scale. The results of the study confirmed the usefulness of nasal allergen provocation testing in diagnosing shrimp allergy [23]. In order to diagnose food allergy in our study, apart from the established diagnostic method (DBPCFC) we used an objective method of assessing nasal mucosal reactivity and demonstrated its usefulness and safety. Importantly, optical rhinometry was used for the first time in assessing the results of an oral food challenge. The objective nature of optical rhinometry increases the reliability of the results. The obtained results suggest that the use of nasal allergen provocation tests may be considered in food allergy diagnostics. Currently, there is a limitation in the form of a lack of standardized allergen extracts for nasal allergen provocation testing.

## Conclusions

The use of an objective method for assessing nasal mucosal reactivity during an oral food challenge increases the reliability of its results and may be its useful complement. Moreover, this opens the door to the possible future use of nasal allergen provocation testing in food allergy diagnostics. However, a more widespread use of this diagnostic technique requires standardization of both the method and the nasally administered allergen extracts.

## Data Availability

Data sharing not applicable to this article as no datasets were generated or analysed during the current study.
